# The dynamics of social networks among female Asian elephants

**DOI:** 10.1186/1472-6785-11-17

**Published:** 2011-07-27

**Authors:** Shermin de Silva, Ashoka DG Ranjeewa, Sergey Kryazhimskiy

**Affiliations:** 1 Department of Biology, University of Pennsylvania, Philadelphia, PA 19104, USA; 2 Uda Walawe Elephant Research Project, 1/657 Thanamalwila Road, Uda Walawe, Sri Lanka; 3 Faculty of Natural Sciences, Open University of Sri Lanka, Nawala Road, Nugegoda, Sri Lanka; 4 Department of Organismic and Evolutionary Biology, Harvard University, Cambridge, MA 02138, USA; 5 Elephant, Forest and Environment Conservation Trust, 215 A 3/7 Park Road, Colombo 5, Sri Lanka

**Keywords:** Elephas maximus, social organization, fission-fusion

## Abstract

**Background:**

Patterns in the association of individuals can shed light on the underlying conditions and processes that shape societies. Here we characterize patterns of association in a population of wild Asian Elephants at Uda Walawe National Park in Sri Lanka. We observed 286 individually-identified adult female elephants over 20 months and examined their social dynamics at three levels of organization: pairs of individuals (dyads), small sets of direct companions (ego-networks), and the population level (complete networks).

**Results:**

Corroborating previous studies of this and other Asian elephant populations, we find that the sizes of elephant groups observed in the field on any particular day are typically small and that rates of association are low. In contrast to earlier studies, our longitudinal observations reveal that individuals form larger social units that can be remarkably stable across years while associations among such units change across seasons. Association rates tend to peak in dry seasons as opposed to wet seasons, with some cyclicity at the level of dyads. In addition, we find that individuals vary substantially in their fidelity to companions. At the ego-network level, we find that despite these fluctuations, individuals associate with a pool of long-term companions. At the population level, social networks do not exhibit any clear seasonal structure or hierarchical stratification.

**Conclusions**

This detailed longitudinal study reveals different social dynamics at different levels of organization. Taken together, these results demonstrate that low association rates, seemingly small group sizes, and fission-fusion grouping behavior mask hidden stability in the extensive and fluid social affiliations in this population of Asian elephants.

## Background

Determining the ecological conditions that shape group formation and social structures is a prerequisite for understanding social evolution [1-4]. Studies in numerous group-living species that follow individuals longitudinally over multiple years find that relationships among individuals change both quantitatively and qualitatively over time [5-9]. This is especially true of societies structured by fission-fusion processes, in which associations among individuals may change over time scales ranging from hours to months [10-13]. Patterns in these dynamics may shed light on the underlying ecological conditions that drive the behavior of individuals [2,3,14,15]. In this paper, we examine social dynamics in a population of Asian elephants.

The longevity and cognitive sophistication of elephants make them potentially capable of maintaining complex social relationships [16-19]. The Asian elephant (*Elephas maximus*), African savannah elephant (*Loxodonta africana*) and African forest elephant, (*Loxodonta africana cyclotis *or *Loxodonta cyclotis*) are the only living members of the proboscidean clade [20-23]. Among adult African and Asian elephants, females and calves form the basis of social units. In African savannah elephants, relatives associate most closely, but multiple social units associate periodically to form hierarchically stratified 'multi-tiered' societies with fission-fusion dynamics [6,24,25]. Relatedness decreases at higher order 'tiers' [26]. Much less is known about the social organization of African forest elephants, but it appears they generally tend to form smaller social groups than savannah elephants [27,28].

Surprisingly little is known of the social behavior of Asian elephants in the wild, despite their long history of co-habitation with people. While it has been commonly assumed that Asian and African elephants behave similarly [29], independent field studies in India and Sri Lanka suggest that adult females associate only with maternal relatives [30,31] and also tend to form smaller groups than African savannah elephants [30,32]. Early studies report that Asian elephants form 'loose' associations with one another, implying unstable social affiliations [32,33]. A more recent study of Asian elephants at Uda Walawe and Yala National Parks in southern Sri Lanka found that matrilineal kin in fact associated together only 18-20% of time, based on observation and telemetry data, leading to the conclusion that individuals from different matrilines are unlikely to associate at all, and that inter-group transfer of females most likely does not occur [30]. It was proposed that family groups fission into daughter groups which then become largely independent of one another [30].

In this paper we investigate how local ecological conditions influence the social dynamics of adult female Asian elephants by tracking associations within a single wild population that inhabits a highly seasonal environment in Sri Lanka. There are at least three distinct temporal patterns of associations that we could potentially observe: random, cyclic, or stable. In the light of previous findings [30], we expect to be able to reject the null hypothesis of random associations. We expect to observe cyclicity in associations if seasonal resource abundance governs the degree to which individuals associate. Alternately, individuals may form stable associations that are independent of environmental conditions. We take cross-sectional and longitudinal views to determine which patterns emerge at different levels of organization within the society. First, we characterize how the strength of the relationship between pairs of individuals changes over time. We then quantify how close companions of individuals, their putative 'ego-networks,' change over time. Finally, we take a bird's eye view of the population to examine whether this Asian elephant society has a tier structure akin to that of African elephants.

### Results

We identified 286 adult females from September 2006 to December 2008. Key terms used throughout the paper are given in Table 1. The number of sightings per identified individual ranged between 1 and 48, with a median of 9. The number of identified adult females seen per month tended to peak at the end of dry seasons in 2007 and 2008 (Table 2), just prior to the onset of the long monsoon, and the rest of the year remained above 66 individuals per month. On average we identified 64% of all adult females encountered. The identification rate was higher (84%) for groups where at least one adult female was previously identified, which is comparable to other similar studies [12]. Group sizes, in terms of the number of adult females encountered in a group, ranged widely but the median was between 2 and 3 in all seasons.

**Table 1:** Glossary of terms.

Term	Definition
Group	A set of individuals observed in the field moving, resting, or interacting non-aggressively within an approximately 500 m radius of one another.
Cluster	A set of individuals that repeatedly associate together such that they form a distinct unit which is revealed by an objective analytical clustering method.
Ego-network	The set of individuals that the central individual, called 'ego', is directly connected to.
Social unit	A general term describing sets of socially affiliated individuals. Social units can form at different levels. For example, an individual's ego-network, a social unit at one level, may be embedded in a larger network, constituting a social unit of a higher level.

**Table 2:** Data summary.

	2007			2008	
	Jan - Apr	May - Sept	Oct - Dec	May - Sept	Oct - Dec
Season Label	T1	D1	W1	D2	W2
No. of individuals	161	201	170	165	154
Observation days	48	68	44	62	37
Mean group size^1^	3	3	2.8	3	2.9
Max. group size^1^	12	13	12	17	10
Mean SRI	0.015	0.011	0.012	0.011	0.013
SD SRI	0.092	0.071	0.07	0.062	0.08
% Non-zero SRI	4.5	4.4	5.3	5.8	4.4
Mean non-zero SRI	0.34	0.25	0.23	0.19	0.29
Number of clusters^2^	24	24	18	18	15
Mean cluster size^2^	6.7	8.3	9.4	9.2	10.3
Max cluster size^2^	29	29	44	44	23

^1^Group size here refers to the number of adult females in a group after data aggregation (see Methods). ^3^Clusters are obtained using the Girvan-Newman algorithm with the SRI threshold equal to zero (see Methods). Distributions of group sizes and cluster sizes are shown in Figure 6.

### Structure at the level of dyads

We measure associations between pairs of individuals using the Simple Ratio Index or SRI [34-36], which is in essence a proportion of time two individuals spend together (see Methods). Summary data are given in Table 2, the SRI matrices and a measure of uncertainty of the SRI values are shown in the supplementary material (see Additional file 1: Figure S1). We find that associations among adult females within all five seasons are significantly nonrandom (sample sizes as in Table 2 permutation test, *P *< 0.001, see Methods). The SRI matrices are also significantly correlated across seasons (Table 3). Thus, pairs of individuals that associate in one season are likely to associate in other seasons and pairs that do not associate in any one season rarely associate in any other season. However, SRI matrices from corresponding seasons across years are not correlated any more or less than matrices from adjacent seasons.

**Table 3:** Correlation between matched SRI matrices.

Comparison	*R*	*N*
T1 vs. D1	0.60	130
T1 vs. W1	0.61	117
D1 vs. W1	0.67	144
D1 vs. D2	0.66	143
D2 vs. W2	0.70	125
W1 vs. W2	0.72	122

*R *denotes the Pearson correlation coefficient. *N *denotes the number of individuals common to both seasons. All *R *values are highly significant (Mantel test, *P *< 0.001).

**Figure 1:**
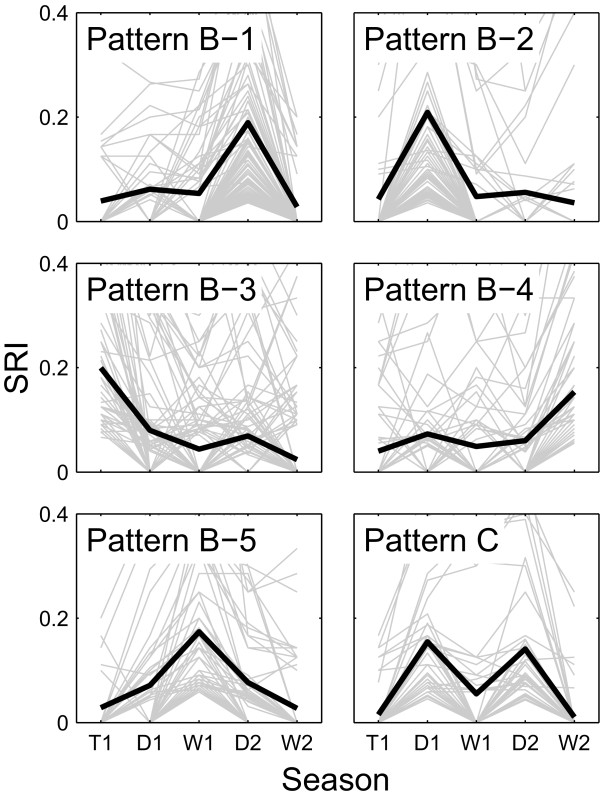
**Typical temporal SRI trajectories**. Typical trajectories are based on K-means clustering of dyadic association vectors, as described in Methods. Light gray curves represent SRI trajectories for specific dyads, heavy black curves show the mean profile for each K-means cluster. Patterns B-1 through B-5 represent associations that peak in a single season (see text). Pattern C represents cyclic associations that peak in dry seasons. Pattern A is shown in Additional file 2: Figure S2. The numbers of individuals and dyads that have each type of the SRI trajectories are given in Table 4.

A simple correlation analysis captures only very coarse features of data and misses more subtle patterns. We therefore examine the social dynamics of the Uda Walawe population in more detail using other methods. In order to maximize the signal to noise ratio in the data, we restrict the subsequent analyses to 51 core individuals, i.e., those that were present in all seasons and were seen at least 30 times during the study period. We plot the SRI values for each pair of individuals as a function of time to determine whether such *temporal SRI trajectories *follow any of the three natural patterns that we expect *a priori *(see Methods for details): stable, which are signified by a flat trajectory (type A), temporary, signified by a single-peak trajectory (type B), or cyclic, signified by a trajectory with multiple peaks (type C). We find that out of the 478 dyads that associated in at least one season, 6 dyads (1.3%) formed by 9 (17.6%) individuals maintain bonds with strength at or above 0.3 in all seasons (Table 4; also see Additional file 2: Figure S2). Thus, approximately 18% of the females engage in at least one relationship that is relatively strong compared to the majority of ties. Although these relationships still fluctuate over time, we consider them to be instances of type A trajectories. Using K-means clustering, we find that the remaining 472 dyads have one of six distinct SRI trajectories, (Table 4, Figure 1; also see Additional file 3: Figure S3). 433 (90.6%) of dyadic relationships (the five most abundant trajectory types) have SRI trajectories of type B, with a single peak in one of the five seasons. All of 51 examined individuals participate in such relationships. Moreover, for 292 dyads (61.1%), the SRI peaks in either the transitional or dry seasons. Finally, 39 (8.2%) of dyadic relationships formed by 32 (62.7%) individuals have the SRI trajectories of type C with peaks in the two dry seasons. This suggests cyclicity in associations among some individuals that a statistical test of correlation across seasons does not pick up. Interestingly, even though single peaks (type B) occur in wet seasons, we do not detect a typical trajectory with peaks in *both *wet seasons.

**Table 4:** Temporal SRI trajectories for the core individuals.

Trajectory type	Peak season(s)	Number of dyads (percent)	Number of individuals (percent)
A	N/A	6 (1.3)	9 (17.6)
B-1	D2	108 (22.6)	39 (76.5)
B-2	D1	99 (20.7)	39 (76.5)
B-3	T1	85 (17.8)	43 (84.3)
B-4	W2	71 (14.9)	43 (84.3)
B-5	W1	70 (14.6)	44 (86.3)
C	D1 and D2	39 (8.2)	32 (62.7)

The total number of potential dyads is 1275, of which 478 (37.5%) had a nonzero SRI value in at least one season. 'Trajectory type' denotes the K-means curve type that best describes the SRI trajectory of the dyad (see Methods), with corresponding plots shown in Figure 1. 'Peak season' refers to the time period in which association strength is greatest. 'Number of dyads' shows the number of dyads whose SRI trajectory has the respective type (percentages are calculated with respect to 478 dyads with at least one non-zero SRI). 'Number of individuals' indicates the number of individuals forming the respective dyads (percentages are calculated with respect to 51 core individuals).

**Figure 2:**
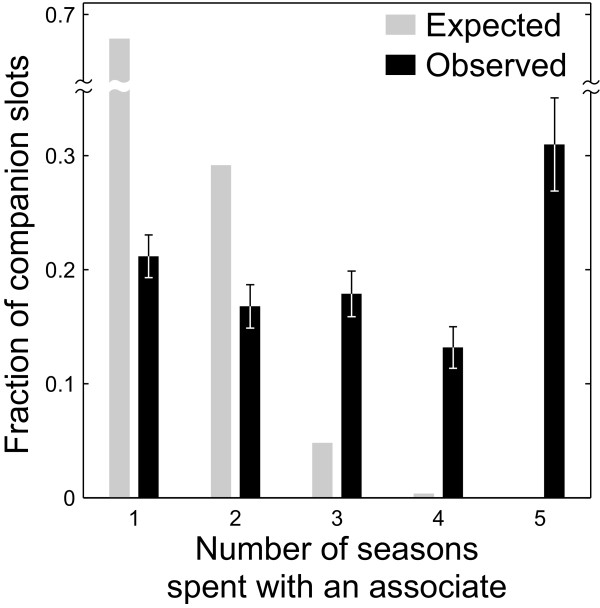
**Allocation of an individual's companion slots to her associates**. The histogram shows the fraction of an individual's companion slots that she allocates to spend with her *m*-term associates, where *m *is shown on the *x*-axis (see Methods for details). Black bars represent the observed mean values, and error bars indicate ± 1 standard error (*N *= 51). Gray bars represent the distribution expected if individuals chose their companions randomly every season.

So far we have been concerned with how the *strength *of association among dyads changes over time. Yet an individual may uniformly increase or decrease the absolute amount of time she spends with others without changing the *rank order *of her preferred companions. We now ask whether the identities of her preferred companions change over time, irrespectively of the tie strength (see Methods for details). We find that a typical individual partitions her time approximately equally among her long-term companions, i.e., those that are present in her top-five for five seasons (21.1% of her time), and her short-term companions, i.e., those that are present in her top-five for one season (31.0% of her time, Figure 2; Wilcoxon signed-rank test, *W *= 480, *N *= 51, not significant). This is different from an expectation under the null hypothesis that individuals randomly reshuffle their companions every season (Figure 2). The observed fraction of long-term companions is significantly higher than the fraction expected under this null hypothesis (observed: 31.0%, expected: 0.01%; *P *< 0.001). Interestingly, individuals have a significantly smaller fraction of medium-term companions, i.e., those that are present in top-five for four seasons, than either short-term companions (Wilcoxon signed-rank test, *W *= 322.5, *N *= 51, *P *< 0.05) or long-term companions (Wilcoxon signed-rank test, *W *= 215.5, *N *= 51, *P *< 0.001), resulting in a U-shaped distribution of companionship preferences (Figure 2).

**Figure 3:**
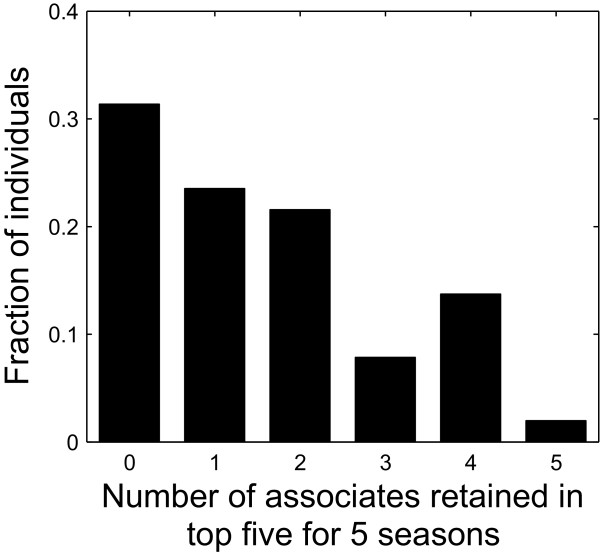
**Turnover of an individual's closest associates**. Bars show the fraction of individuals that retain a given number of associates in their top-five (see Methods and Results). Value zero in the *x*-axis implies that the identities of an individual's top-five associates have completely changed over five seasons; value 5 implies that her top-five associates have not changed over this period. For example, approximately 20% of individuals retained two associates over all five seasons.

**Figure 4:**
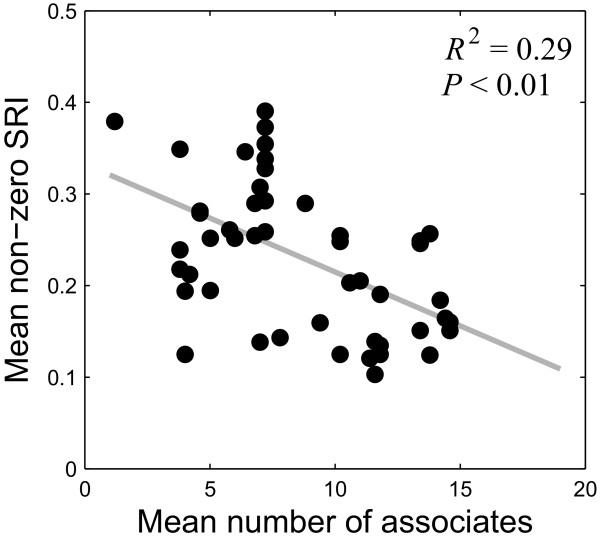
**Mean SRI of an individual versus the number of her associates**. Each point shows the number of associates of an individual averaged over 5 seasons and the SRI value of the individual averaged over all of her associates and all seasons (see text for details). Data for each season as opposed to averages over all seasons are shown in Additional file 4: Figure S4.

We also observe individual variation in social behavior at the level of dyads. 8 females (15.7%) maintained 4 to 5 of their top-five companions for all five seasons, while 16 females (31.4%) completely changed their top-five companions over the course of the study (Figure 3). Moreover, individuals vary by as much as factor of 4 in the number individuals they associate with on average within a season and in the average strength of their ties (Figure 4). We also observe that those individuals who associate with many individuals (i.e., those that have many non-zero SRI values) typically have weak ties to their associates (mean non-zero SRI is low), whereas those who maintain few associates have strong ties to them (Figure 4, negative correlation, *R*^2 ^= 0.29, *P *< 0.01; for data by season rather than averages see also Additional file 4: Figure S4). Since the association index reflects the amount of time two individuals spend together, this negative correlation suggests a trade-off between the number of associates that an individual can maintain and the amount of time she can spend with each of those associates.

### Ego-network structure

**Figure 5:**
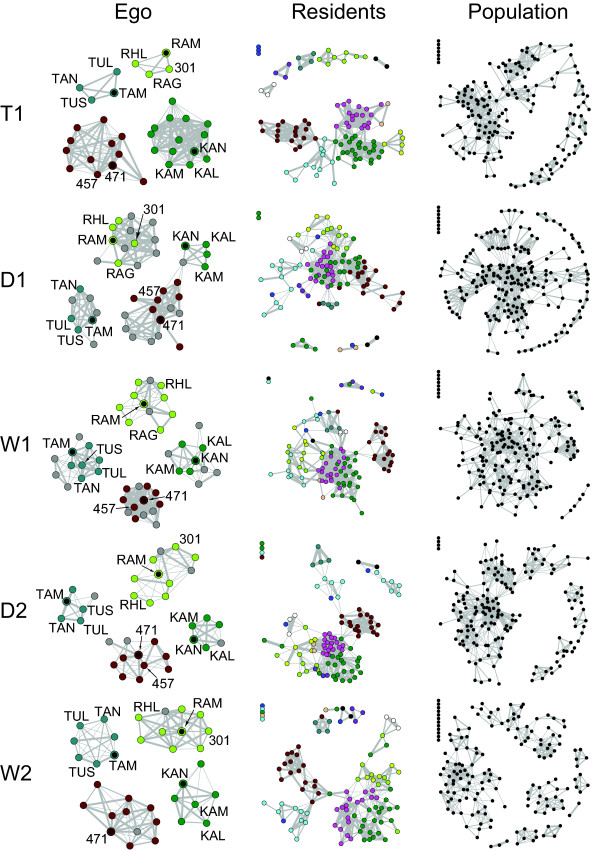
**Social networks by season**. Nodes represent adult females and the thickness of edges corresponds to the SRI value. Isolates appear in the upper left corner of each network. Each row corresponds to a season and each column to a type of network. 'Ego' shows the ego-networks of selected subjects, who are indicated by black circles with colored borders. Node colors correspond to each Ego's Girvan-Newman cluster assignments in T1. These colors are maintained through all seasons to allow comparison across seasons (actual cluster designations in other seasons are not shown). In subsequent seasons, individuals colored gray are those who did not appear as Ego's companions in any preceding season covered by this study, although they may have associated prior to January 2007. Labeled nodes indicate those who associated with the subject in nearly every season. By the fifth season, networks clearly consist primarily of individuals who previously associated with the subject, even if not all were present in every season. 'Residents' shows all residents. One can track the coherence of each cluster over time. Some clusters from T1 maintain their integrity (e.g. brown) whereas others do not (e.g. light blue); associations among clusters also change over time, most notably the large pink and dark green clusters. Clusters are connected via just a few bridging individuals. 'Population' shows the full social network for each season with sample sizes reported in Table 2. Social networks constructed from real data have distinct structure, whereas those constructed from randomized data do not (see Additional file 5: Figure S5).

We now expand our view beyond dyads to look at multiple direct associates of an individual simultaneously in a larger sample of 88 residents, i.e., individuals that were observed in every season (see Methods). We represent SRI matrices as weighted social network graphs [37,38], where nodes correspond to individuals, edges connect those who have been associated within a season, and edge weights correspond to the SRI values (Figure 5). An ego-network is then a social network that consists only of the subject, or 'ego,' and the nodes to which she is directly connected, i.e. individuals with whom she was associated at least once (Table 1). For each resident, we compute five ego-network measures, defined in Table 5. Average values of ego-network measures over all residents are called ego-network statistics.

**Table 5:** Ego-network statistics.

		Size	Ties	Pairs	Density	2-Step Reach
T1	Mean	8.6	30.7	51	0.77	23.2
	SD	6.1	32.4	63.9	0.23	16
	BM^1^	7.1	23.6	35.4	0.82	16.7
D1	Mean	12.6	55.5	100.4	0.7	41.1
	SD	7.5	48.9	114.7	0.21	23.8
	BM^1^	10.2	40.3	68.3	0.74	30.7
W1	Mean	11.4	45.2	74.1	0.71	36
	SD	5.5	34.6	71.9	0.2	19.5
	BM^1^	8.7	29.7	45.2	0.76	23.7
D2	Mean	12.9	63.7	106.9	0.72	40.6
	SD	7.8	66.9	127.3	0.19	24.3
	BM^1^	10.4	46	73.6	0.74	30.2
W2	Mean	7.5	19.8	31.9	0.75	20.7
	SD	3.8	18	35.3	0.22	10.9
	BM^1^	6	14	20.7	0.79	14.1

^1^BM is the mean of the bootstrap distribution

'Size' is the number of the subject's (ego's) direct companions, i.e., those individuals seen in a group with her at least once. 'Ties' is the number of ties between a subject's direct companions. 'Pairs' is the number of potential ties in the ego-network, i.e., the number of ties that would exist if all of subject's direct companions were also each other's direct companions. 'Density' is the ratio of actual to potential connections in the ego-network (Ties/Pairs), indicating how well-connected companions are to each other. '2-Step Reach' is the number of individuals within two degrees of separation from the subject, i.e., the number of friends and friends of friends. For each season, the first column shows the ego-network statistic, i.e., the mean of the distribution of ego-network measures across 88 residents; the second column shows the standard deviation of this distribution, and the third column shows the expected value of the ego-network statistic derived from 1000 bootstrap samples. Bootstrap distributions were generated as described in Methods. The bootstrap distributions for the Size, Ties, Pairs and 2-Step Reach statistics are shifted towards lower values with respect to the actual values in all seasons, and the bootstrap distribution of the Density statistic is shifted towards higher values in all seasons. All bootstrap distributions are significantly different from each other across seasons (Wilcoxon rank sum test, *P *< 0.001).

All ego-network statistics except 'Density' tend to have lower values in wet seasons than in dry seasons (Table 5), indicating that ego-networks are larger in dry seasons than in wet seasons. The measure 'Density' is marginally lower in dry seasons, implying that the ego-networks are less interconnected in dry seasons than in wet seasons. Because ego-network measures for different individuals are non-independent, we cannot directly test for differences between the distributions of ego-network measures in different seasons. Instead, we test for differences in the bootstrap distributions of ego-network statistics [39]. The bootstrap means of ego-network statistics are reported in Table 5. Bootstrap distributions for all ego-network statistics differ significantly between seasons. We note however that these distributions for all statistics in all seasons are systematically shifted with respect to the observed values (Table 5). This suggests that the bootstrap method we used produces biased distributions of ego-network statistics, but we are not aware of a better method for statistical comparison. We therefore must be cautious with the interpretation of Table 5.

The ego-network measures are sensitive to the number and arrangement of companions but ignore their identities. However, visualizations of ego-networks demonstrate that while a subject's direct companions do change over time, she has a few that are almost always present; even those that are not present continuously may have been companions in previous seasons (Figure 5). Thus, individuals maintain long-term relationships with others even though they may be apart for one or several seasons and have low SRI values.

### Population-level structure

**Figure 6:**
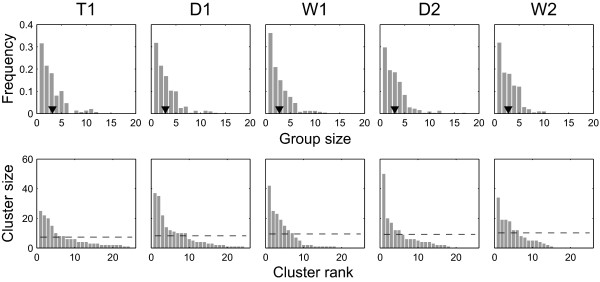
**Group- and cluster-size distributions by season**. Top row shows the group size distributions. Inverted triangles show the mean group sizes (see Table 2). Bottom row shows the size of Girvan-Newman clusters ranked by size. The horizontal dashed lines show the mean cluster size (see Table 2).

**Figure 7:**
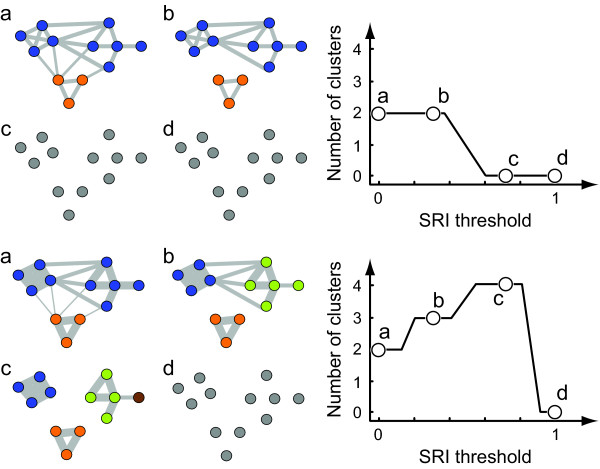
**Schematic social networks and corresponding network structure curves**. Top and bottom panels show two networks that have identical topology, but different distributions of tie strengths. The network in the top panel has a homogeneous distribution of tie strengths while the network in the bottom panel shows a heterogeneous distribution. Edge widths indicate tie strength. The Girvan-Newman clustering algorithm detects highly interconnected subunits regardless of edge weights, and so, at the SRI threshold of 0 both networks have the same number of clusters (a). By removing ties with the SRI values below successive thresholds and re-running the clustering algorithm, the underlying differences in the networks are easily visualized in the network structure curve on the right (b-d).

**Figure 8:**
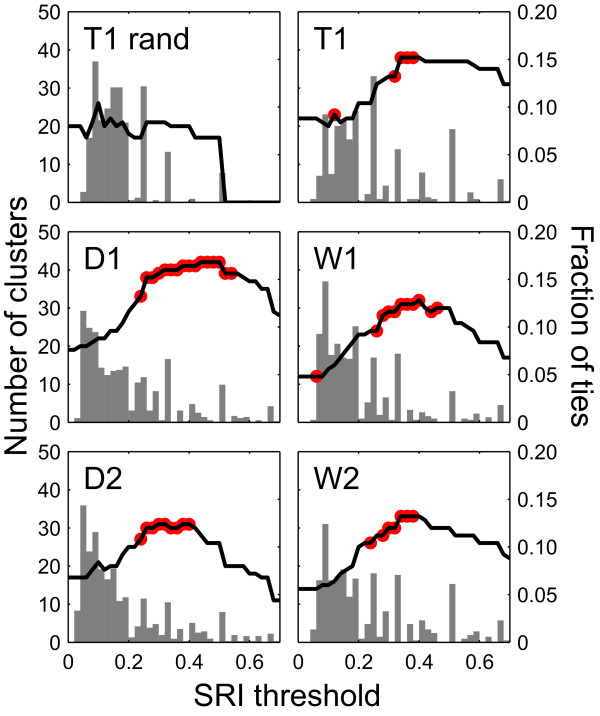
**Network-structure curves and SRI distributions by season**. Black curves show the number of Girvan-Newman clusters in the thresholded network (left *y*-axis). Gray bars show the fraction of dyads with the given SRI value (right *y*-axis). Red dots show the points at which the network-structure curves display slope changes at the significance level of 0.05 or below with the window size *w *= 0.2 (see Methods; exact *P*-values for this and other window sizes are shown in Additional file 8: Figure S7). While the majority of ties are weaker than 0.2 in both real and randomized data, the distribution has a long tail in real data but not in randomized data. Randomized network shows no ties above a threshold of 0.5, and a nearly level network structure curve up to this threshold with no significant slope changes. In contrast, observed networks for each season show peaked curves with significant changes in slope. *Q_max _*remains above 0.6 for real data while for randomized data it peaks and falls sharply (see Additional file 9: Figure S8).

We now step out further and examine global relationships among the entire study population within each season using the Girvan-Newman method for finding community structure, as described in the Methods. To avoid confusion with sociological definitions of communities, here we refer to the subnetworks found by this algorithm simply as 'clusters.' These clusters represent one or more social units, i.e. sets of individuals that have more ties to one another than to those outside of the set (Table 1). Results are presented in Figures 6, 7 and 8.

First, we note that the social network formed by individuals in this population is extensive and well-connected (Figure 5). Moreover, in all seasons, a typical cluster detected by the Girvan-Newman algorithm is 2 to 3 times larger than a typical group encountered by observers in the field (Figure 6 and Table 2). These findings are unexpected in the light of previous studies which have suggested that Asian elephants do not display extensive social affiliations [30,33].

Next, we examine how the structure of the social network changes over time. For each season, we plot the network structure curves (Figures 7 and 8; see Methods for detailed explanation), which show how ties of various strengths are distributed in a manner that is easier to digest than the social network diagrams depicted in Figure 5 (the effect of the thresholding procedure on the T1 network is illustrated in Additional file 5: Figure S5). A network generated from randomized data shows no distinct clusters (see Additional file 6: Figure S6). As a result, the corresponding network structure curve remains flat until the SRI threshold reaches the mean tie strength in the network, at which point the curve rapidly declines to zero (Figure 8). In contrast, the network structure curves for real data have a distinct peak. This is probably a consequence of the fact that both extremely weak ties and extremely strong ties can be influenced by sampling effects, but intermediate values clearly distinguish core social units. Although all seasons have 10-20 well-defined clusters at the SRI threshold of zero, network structure curves for each season behave differently at subsequent SRI threshold values. In particular, the slope of the curve changes only once (from positive to negative) in seasons D1, D2 and W2, but changes twice (from zero to positive and from positive to negative) in seasons T1 and W1 (Figure 8). This suggests that the network structures in each season are different (also visible in Figure 5). Although the network structure curves are different across seasons, there appear to be no patterns characteristic of dry or wet seasons *per se*. Visual inspection of networks shows that, while many clusters maintain their integrity across seasons, some individuals transfer between clusters, and the connections among clusters change (Figure 5).

## Discussion

We have presented the first detailed quantitative characterization of social organization in an Asian elephant population, at multiple levels of organization and across ecological timescales. We asked whether associations are random, stable, or cyclic. We find that the answer depends on the timescale and level of organization. Most ties are weak (SRI values below 0.3) compared those of African savannah elephants where typical association rates are above 0.6 [6,13,25]. Despite the overall weakness of ties, most individuals have a few strong ties (SRI values exceeding 0.3) as well as a few consistent ties (maintained over several seasons) with some of their associates. Individuals do not mix randomly within the population, nor are they always with the same companions, but rather they shuffle amongst a subset of preferred companions. All individuals engage in temporary associations, especially during dry seasons. Some also associate to a greater degree in dry seasons, forming cyclic associations. This cyclicity is evident at the level of dyadic associations (Figure 1), but not at the ego-network or population levels.

Our results suggest a view of Asian elephant social structure that is different from what has been described in the literature before. Earlier studies reported small group sizes typically consisting of less than five adult females [30,33], a number comparable to the group sizes observed in this study. The number of individuals with the same mitochondrial haplotype ranged from 3 to 12 [30], which could be taken as matrilineal family sizes. However, families and observer-defined groups are only relevant structural units insofar as they relate to actual *social units*, expressed through the animals' behavior. The latter are revealed by a quantitative analysis of long-term association data rather than genetics [6,26]. It took up to two years to obtain repeated observations of all members of some social units in this study (Figure 5 and unpublished data from 2005-2006). In this paper, we used the Girvan-Newman procedure to detect such social units and found that they are in fact much larger than either the group or family sizes reported in previous studies (Table 2, Figures 5 and 6). Such units are more stable across years than individuals' immediate companions. Informal photographic records suggest that some of the individuals in this study have associated since at least 2001 (unpublished data), which supports the existence of long-term stable associations. Since Asian elephants are capable of communicating both chemically [29,40] and acoustically [41,42] at distances humans find difficult to observe, they may be aware of the their associates' locations even when the latter are beyond the visual range of human observers. Indeed, vision is not the preferred mode of perception for elephants, as we see individuals track precise paths taken by others using scent even when both parties are plainly visible to humans. Moreover, outside protected areas, elephants are largely nocturnal (personal observations). 'Groups' of Asian elephants are not unlike those formed by African elephants [13]. As with other animal societies that exhibit fission-fusion dynamics, such as that of chimpanzees [43,44], the social organization of a highly mobile species like Asian elephants is not fully evident without systematic and prolonged observations, particularly in areas where visibility is restricted. Long-term observations of other Asian elephant populations would be extremely useful to corroborate this finding.

Previous authors also concluded that associations among different families were highly unlikely as associations even among family members appeared infrequent [30]. However, these conclusions were drawn from extremely small sample sizes (for instance, only 1 mtDNA haplotype from Uda Walawe and few repeat observations). Our results suggest otherwise. While the association rate of 18-20% reported in a previous study [30] is roughly analogous to the median SRI value of found in our study (Table 2, Figure 6), we do find reliable SRI values that range as high as 1 (see Additional file 1: Figure S1). Moreover, there is at least some transfer of individuals between social units across seasons (Figure 5). However, the population-level social network structure does not appear to exhibit any clear seasonal patterns (Figure 8). It is not clear whether individuals form hierarchical social 'tiers', such as those observed in African savannah elephants, which form higher-order associations among multiple families in wet seasons [6,24,25,45]. Among savannah elephants, relatedness decreases at higher-order tiers of association, where associations are weaker than 0.6 [13,26,46], and are mediated by intra- and inter-group dominance interactions [47,48]. The network structure curve for Asian elephants peaks near an SRI threshold of 0.3 (Figure 8). Although there is no consistent social stratification in this Asian elephant population, it is possible that the clusters prior to the peak have a lower degree of relatedness than the clusters that follow it. A detailed genetic study of this population examining the hypothesis above, with larger sample sizes than previously obtained, would also be illuminating and is planned in the future.

There is much variation in individuals' long-term fidelity to companions (Figures 3 and 4). For instance, Kamala (KAM) and Kanthi (KAN) were two mature females who appeared close to the same age and were nearly always together (Figure 5). The so-called 'K' unit (Kamala, Kanthi, Karin, Kavitha and Kalyani) almost always contained every member whenever it was seen although they also interacted with others to form a larger cluster. On the other hand, individuals like '471,' also part of a large cluster, had few stable companions (Figure 5). The social placement of a few other females remained unresolved despite numerous repeat observations. The fitness consequences of these different social strategies remain to be seen. Moreover, while it is widely assumed that Asian elephants, like African elephants, form strongly bonded family groups centered around matriarchs [29,40], the apparent variation and fluidity in social preferences shown in this study would seem to question such a characterization.

It is intriguing that social dynamics differ depending on the level of analysis - the bottom-most (dyadic) and top-most (population) levels of organization exhibit a greater degree of instability than the intermediate level (social units and long-term ego-networks). Uncovering the ecological basis for observed patterns would require separate investigations and hypotheses at each level. Preferences for one another shown by some pairs of individuals might depend on reproductive state (e.g. those with similarly-aged calves), while social units may differ in their strategies depending on whether they are seasonal inhabitants of the park or residents. For instance, killer whales of the same species exhibit different social strategies depending on whether they are resident or transient, in accordance with the associated feeding ecology [5].

Our analyses are based on association index data, calculated from observations of individuals in the field. There are at least two sources of uncertainty associated with this type of data. First, we expect that some variation comes from the fact that we observe different sets of individuals in different seasons. Our observation area is largely constrained by the road network inside Uda Walawe National Park. While we expect to have a reliable observation record for individuals whose range strongly overlaps with this area, we also observe individuals that presumably move into this area only periodically [49]. Such individuals, being farther away from the centers of their home ranges, might exhibit different behavioral and social patterns than individuals residing more centrally, thus introducing additional noise in our data. The second source of variation stems simply from relatively low counts of events in some seasons for some individuals. To minimize the first type of noise, we have constrained most of our analyses to the so-called resident individuals, i.e. those that we have consistently observed every season. To minimize the second type of noise, we have constrained some of the analyses even further, to individuals that have been seen at least 30 times. Nevertheless, we can extrapolate the conclusions drawn from these analyses to less sampled individuals in the population, since such individuals are not ostensibly different from those sampled thoroughly. One does however need to keep in mind that we describe the behavior of individuals that are close to the center of their home range.

One of our surprising findings is that the elephants at UWNP tend to form a greater proportion of strong ties in dry seasons than in wet seasons. This suggests that aggregation may be more advantageous in the former, perhaps for accessing and protecting scarce resources. This hypothesis remains to be tested with additional seasons of data and behavioral studies. While direct behavioral evidence of resource defense among adult females is rare, we have observed competition over water and mud, dominance interactions when unfamiliar individuals or social units meet, as well as the vocal and physical displacement of one social unit by another [41]. Resource monopolization may more often take the form of competitive exclusion rather than confrontation, in which acoustic and chemical signals facilitate social cohesion as well as avoidance despite the seeming fluidity of associations. Herbivores must balance intraspecific resource competition against potential anti-predator benefits [3,50,51]. Among artiodactyles, gregariousness is an anti-predator adaptation seen in species inhabiting open environments [52]. African savannah elephants likewise may be more gregarious than Asian elephants because they typically inhabit more open environments, and also encounter predators other than humans [53-55]. Interestingly, in drier regions of Sri Lanka just a few kilometers east of the study site, elephants are reported to aggregate in wet seasons rather than dry seasons [56], a similarity to African savannah elephants that could be ecologically driven. Similar longitudinal studies in other Asian elephant populations, especially those in India, where there is likely to be greater variation in habitat quality and home range sizes [57] would be of great interest. More data are also needed on African elephants occupying various habitats including desert and forest environments, the latter being more similar to those of many Asian populations. It is possible that societies in general and elephant societies in particular are more flexible and responsive to environmental pressures than generally conceded.

## Conclusions

Associations among female Asian elephants can be characterized as fission-fusion. Patterns of association differ across levels of organization and ecological timescales. Individuals are found by observers in small groups whose composition changes on the timescale of days. However, most individuals belong to relatively large social units which can be revealed by quantitative analysis of longitudinal data. Such units generally maintain their cohesiveness over longer timescales, although there is some transfer of individuals between units. Social units may fission or fuse without discernible seasonal patterns or clear hierarchical stratification into social 'tiers' at the level of the population. Individuals vary highly in the number of stable companions they maintain over time, and some repeatedly associate in dry seasons. Their companions tend to form a pool of long-term associates. Interesting future directions include examining the relatedness among social units, the ecological basis of observed dynamics at each level of organization, and the fitness consequences of the seemingly different social strategies employed by individuals.

## Methods

### Study site

Uda Walawe National Park (UWNP), Sri Lanka, is located between latitudes 6° 25' - 6° 34' N and longitudes 80° 46' - 81° 00' E, at an average altitude of 118 m above sea level. It encompasses 308 km^2 ^around the catchment of the Uda Walawe reservoir. The study area comprises approximately 1/3 of this area, which includes tall grassland, dense scrub, riparian forest, secondary forest, a permanent river, seasonal streams, and other water sources. It has a highly predictable pattern of rainfall with two monsoons per year, which occur in March through April and in October through December [58,59]. There appear to be no non-human predators of elephants at this location. The Sri Lankan subspecies of Asian lion (*Panthera leo sinhaleyus*) was extinct prior to the colonization of the island by humans [60]. The leopard (*Panthera pardus cotiya*) is the current top land predator in Uda Walawe, but there is no evidence that it hunts elephants. The only other large predator is the freshwater crocodile (*Crocodylus palustris*), but predation on elephants has not been documented. The greatest threat to elephants both historically and currently is human activity, but disturbance within the park is minimal. Tourism has also led to the elephant population becoming well-habituated to people in vehicles.

### Data collection

The data presented here span 259 field days (twenty months) from years 2007 and 2008, two or three days per week on average except between January and April 2008, when UWNP was temporarily closed due to political unrest. We typically entered the park between 0600-0700 h (sunrise), remaining continuously inside until 1730-1830 h (sunset). Driving routes were varied such that all accessible parts of the park were covered in a week. Locations where animals were closest to the road were marked on a hand-held GPS unit. Temperature, humidity and wind were recorded at least three times per day with a Kestrel™ pocket weather station. Rainfall (mm.) was recorded daily using a standard U.S. Weather Bureau rain gauge.

Individuals were identified photographically and catalogued for two years preceding the study period. All individuals were given numbers; the most frequently seen were also given names. A previous study of Asian elephants considered individuals within 100 m of one another to be associated [30] whereas studies of African elephants have used a distance of up to 500 m to define aggregations [6,25,61]. We considered all individuals within visual range of the observer and up to 500 m of one another who moved, rested, shared resources (mud, mineral wells, trees) to be a single aggregation, or group. Occasionally individuals showed affiliative vocal or tactile behavior such as growling and bodily rubbing [41] but such interactions were uncommon and not required for individuals to be considered part of the same group. Multiple groups occasionally shared water without interaction. The term 'group' here carries no implication of social history or permanence (see also Table 1). Individuals from multiple groups which initially co-occurred in space or even passed through one another, were not counted as associated unless they actively moved together. It was possible to spend several hours with a single group. We recorded identities of known individuals and counted the number of individuals in five size-based age classes [49]. Unidentified individuals were counted, but excluded from analyses.

We examined only relationships among adult females, as most sub-adults and juveniles were not identified individually. The strength of one individual's bond with another individual over the course of a season was quantified in terms of their association index. We used the Simple Ratio Index or SRI [6,35,36,62], which is a symmetric measure that shows the proportion of time two individuals spent with each other. Before computing the SRI, we performed data aggregation which consisted of (a) partitioning the data into day-long sampling intervals; (b) identifying individuals that associated with each other in a given sampling interval by merging those groups that shared at least one identified individual; all individuals within such groups were then, by definition, 'associated'; and (c) excluding individuals that were observed only once in a season and only alone. Step (b) was performed in order to exclude potentially non-independent observations of the same group within the same sampling period. After data aggregation, we compute the association index for each pair of individuals A and B as SRI = *X_AB_*/(*X_t _*- *X_n_*) where *X_AB _*is the number of times individuals A and B were observed together, *X_t _*is the total number of observations, and *X_n _*is the number of observations in which neither A nor B were observed [62]. We also compute a measure of uncertainty of the SRI value for each dyad (see Additional file 7: Supplementary Text).

Association data were partitioned according to season. Months that had a total rainfall higher than the two year monthly average of 120 cm were designated as 'wet' months and those that had less were designated as 'dry', consistent with the monsoon cycle [59]. May-September constitute the 'Dry season' and October-December constitute the 'Wet season' according to this classification. January-April, with two wet months followed by two dry months, were considered a 'Transitional' period rather than divided into dry and wet periods since two month periods provided insufficient data for analysis. We refer to seasons as T1, D1, W1, D2 and W2 (Table 2).

### Data analysis

To reduce variance in our data that arises from differences in the identities of individuals observed in different seasons, we constrain some of the analyses below to the set of 88 individuals that were observed in all seasons (either more than once or in association with other individuals). We refer to these individuals as 'residents'. To further reduce noise in the data due to low number of sightings, we constrain some of the analyses to the 51 residents that were observed at least 30 times throughout the entire study period. We refer to these individuals as the 'core individuals'. The uncertainty in the estimates of association indices for such individuals is generally below 10% for all seasons (see Additional file 1: Figure S1).

### Dyadic structure

To test the data against the null hypothesis that associations *within *a season are random, we permute the seasonally partitioned datasets so that the number of sightings for each individual and the distribution of group sizes within the season are preserved. We use the 'fill' rather than the 'swap' method to generate 1000 permutations per season [63-65], with the average SRI value as the test statistic. To speed up computations, we partition the dry seasons into two overlapping three-month periods (May-July and July-September). We use some of the random datasets generated by this procedure in other analyses.

We examine the stability of associations among pairs of individuals *across *seasons in a few different ways. First, we test for correlations between matched SRI matrices across pairs of seasons using the Mantel test [63,66,67]. As not all individuals are seen in all seasons, by 'matched' matrices we mean that for each pair of seasons we test correlations only across the subset of individuals seen in both seasons. We used 10,000 permutations per test, with the Pearson product-moment correlation coefficient as the test statistic. This is the most basic way to test whether associations across seasons deviate from random [68].

Second, we track the SRI for each pair of individuals as a function of time and assess, using K-means clustering, whether such *temporal SRI trajectories *fall into distinct types. Three temporal patterns are natural to expect. If associations are stable, we expect to see a flat SRI trajectory (type A). If associations are temporary, we expect to see an SRI trajectory with a single peak at a particular season (type B). If associations are cyclic, we expect to see an SRI trajectory with more than one peak, in corresponding seasons across years (type C). In order to minimize noise in the data due to rarely observed individuals, we limit this analysis to the 51 core individuals (see above) that can potentially form 1275 dyads. We exclude those dyads that have never associated during the study period, yielding 478 dyads with at least one non-zero SRI value within the study period. We use the correlation distance between SRI trajectories as the metric for K-means clustering because in this analysis we are interested in similarities in the shape of temporal SRI trajectories rather than in their absolute values. The number of clusters, *K*, that is most appropriate for the data, is chosen using the Bayesian Information Criterion (BIC) where each K-means cluster is assumed to be generated by a Gaussian distribution [69]. We perform the K-means clustering procedure 100 times for each value of *K *between 2 and 15, starting with a random initial condition, and choose *K *at which the expected BIC is maximized as the optimal *K*. After determining the appropriate value of *K*, we run the K-means clustering algorithm 1000 times with different initial condition and pick the partition of the SRI trajectories into clusters that minimizes the sum of distances between the SRI trajectories and the cluster centroids to which they belong. In order to avoid confusion with the population-level clustering procedure below, K-means clusters are henceforth characterized by their centroids and are referred to as 'typical SRI trajectories'.

To investigate whether an individual's preferred companions change over time irrespective of the strength of the ties, we determine the top-*n *associates of each core individual within each season. Top-*n *associates of a core individual *i *in the given season are defined as the *n *core individuals (other than *i*) who have the highest SRI values with respect to individual *i *in that season. Then, in each season, a core individual *i*, by definition, allocates *n *'companion slots' of time to spend with her top-*n *associates. Thus, the total number of companion slots available to each individual in 5 seasons is 5*n*. If individual *j *is present in the individual *i*'s top-*n *over *m *seasons (1 ≤ *m *≤ 5), we say that associate *j *'occupies' *m *companion slots of individual *i*, or that individual *i *allocates *m *of her companion slots to individual *j*. We then call individual *j *'an *m*-term associate' of individual *i*. If *m *= 1, individual *j *is a short-term associate of individual *i*, while if *m *= 5, individual *j *is a long-term associate of individual *i*, with obvious gradations in between. We arbitrarily set *n *= 5. To illustrate these concepts, consider two individuals A and B. If B's SRI index with respect to individual A is ranked 3^rd^, 2^nd^, 6^th^, 5^th^, and 9^th ^in seasons 1 through 5 respectively, then B is in A's top-5 associates for 3 out of 5 seasons and therefore occupies 3 companion slots. Thus, individual B is a 3-term associate of individual A.

Now, if *k_i_*(*m*) is the number of individual *i*'s *m*-term associates, then *f_i_*(*m*) = *k_i_*(*m*)*m*/5*n *is the fraction of companion slots that individual *i *allocates to all of her *m*-term associates (note that  because each individual has a total of exactly 5*n *companion slots). Then the average fraction of companion slots that individuals allocate to their *m*-term companions is , where *N *= 51 is the total number of core individuals.  indicates the extent to which individuals prefer to associate with the same companions over many seasons or to change companions from season to season.

To quantify individual variation in social behavior, we count, for each of the 51 core individuals, the number of associates that are present in her top-*n *for the whole observation period for *n *= 5. This number ranges from 0 to *n *= 5.

### Ego-network structure

We represent the SRI matrices as weighted social-network graphs [37,38], where nodes represent individuals, edges connect those individuals who were associated within a season, and edge weights correspond to the SRI values. An ego-network is a social network that consists only of the subject, called 'ego,' and the nodes to which she is directly connected. For each of the 88 residents, we compute five ego-network *measures *with intuitive biological interpretations: Size, Ties, Pairs, Density, and 2-Step Reach (defined in Table 5). We then compute the corresponding ego-network *statistics*, i.e. the average values of ego-network measures over all residents. As ego-network measures for different individuals are non-independent, we test for differences in ego-network statistics between seasons using a bootstrap procedure in which association data for each season are re-sampled 1000 times with replacement [39].

### Population structure

In order to investigate how the social network structure of the whole population changes over time, we construct 'network structure curves' (Figure 7) for each season using the following procedure. First, using the full SRI matrix for each season, we construct the social network graph for the whole population (i.e., we include non-resident individuals in this analysis). We then identify social clusters within the network using the Girvan-Newman algorithm for community detection [70]. This algorithm recursively fragments the network into subnetworks by successively removing the edges with the highest between-ness [37]. For each resulting subdivision of the network, the so-called 'modularity quotient,' *Q*, is computed. The modularity quotient takes values between 0 and 1, where 0 implies that the number of ties within a cluster does not exceed a random expectation, and values above 0.3 indicate potentially meaningful subdivisions [71-74]. We label the maximum modularity for a given social network as *Q*_max _and take the partition or partitions that yield this maximum value to be the most appropriate way of subdividing the network. The term 'cluster' refers then to the set of individuals belonging to the same subnetwork in an optimal subdivision.

The original Girvan-Newman algorithm was designed to find community structure in unweighted networks. To accounts for edge weights, we add one more step. We apply the Girvan-Newman procedure to a social network that consists only of ties with strength above a particular threshold, and record the number of clusters in the resulting network (Figure 7). If multiple subdivisions of the network at a given threshold yield identical *Q*_max _values, we record the average number or clusters (e.g. if either 14 or 15 clusters have an equivalent *Q*_max _at a threshold of 0.1, we record 14.5 clusters). We perform this procedure iteratively for different SRI thresholds, incrementing by 0.02: 0, 0.02, 0.04, ..., 1. We then plot the average number of clusters against the SRI threshold. We call the resulting plot the 'network structure curve,' as it shows the structure of the network at a glance, being a lower-dimensional visual representation than social network graphs (Figure 7).

In order to test whether network structure curves have significantly different shapes in different seasons we modify the method used by Wittemyer et al. [6]. For each SRI threshold value, we compare the distributions of increments of the network structure curve within a window *w *before and after this value, using the Mann-Whitney test. We then find points in the curve where these distributions are different from each other at the significance level 0.05. The points with significant *P*-values are consistent when we vary the size of window *w *between 0.1 and 0.3 (see Additional file 8: Figure S7 and Additional file 9: Figure S8).

### Implementation and ethical statement

Permutations of associations, observed group sizes, and corresponding significance tests within and across time partitions were implemented in the OCaml programming language; code is available upon request. Network visualizations were generated in NetDraw using a graph-theoretic layout with node repulsion [38]. All other statistical procedures and analyses were performed on Matlab v. 7.0, and R v. 2.7. This work was carried out in compliance with requirements of the Institutional Animal Care and Use Committee of the University of Pennsylvania, protocol number 801295.

## Authors' contributions

SdS conceived of the study, supervised fieldwork, collected field data, developed analytical methods, conducted data analyses, and wrote the paper. ADGR participated in planning, running the study, collecting and entering field data. SK developed and implemented analytical methods, performed statistical data analyses, and wrote the manuscript. All authors read and approved the final manuscript.

## Supplementary Material

Additional File 1 **Figure_S1.pdf. Association index matrices and matrices of uncertainty by season**. SRI matrices (left column) and the matrices of uncertainty measure based on expression [S1] in the supplementary text (right column). See additional File 7 for supplementary text. *x- *and *y- *axes represent individuals, ordered by the total number of sightings across the whole study period. Red squares show the individuals with 30 or more sightings. Click here for file

Additional File 2 **Figure_S2.pdf. Temporal SRI trajectories of type A**. Type A trajectories are relationships that are maintained at SRI values above 0.3 in all seasons, and hence exemplify stable associations. Click here for file

Additional File 3 **Figure_S3.pdf. Determining the optimal number of clusters for clustering temporal SRI trajectories**. Bayesian Information Criterion (BIC) as a function of the number of clusters, *K*, into which the temporal SRI trajectories are partitioned by the K-means algorithm. Light gray curves represent 100 K-means clustering runs with random initial conditions. Thick black curve represents the mean over these runs. The mean BIC curve peaks at K = 6 indicating that there are 6 typical shapes of temporal SRI trajectories. Click here for file

Additional File 4 **Figure_S4.pdf. Trade-off between the number of associates and the strength of association by season**. Each point shows the number of associates of a core individual (abscissa) and the average non-zero SRI value for that individual (ordinate). Data is displayed for each season separately (averages across seasons are shown in Figure 4 in the main text). Click here for file

Additional File 5 **Figure_S5.pdf. Girvan-Newman clusters for season T1 at various SRI thresholds**. This figure is analogous to the schematic shown in Figure 7 in the main text. Corresponding network structure curves are shown in Figure 8 of the main text. Individuals that do not have ties at or above the threshold value are removed for the sake of clarity. Arrows indicate groups enlarged in the last two panels. Cluster colors correspond to Figure 5 in the main text. Maximum separation of groups occurs in this season at SRI values above 0.4. Beyond the SRI threshold of 0.6 most of these clusters also degenerate. Click here for file

Additional File 6 **Figure_S6.pdf. Social network for randomized T1 data**. An example of a social network generated by the randomization procedure described in the methods. The network is relatively homogenous, without distinct clusters. Click here for file

Additional File 7 **Supplementary_Text.doc**. Description of the procedure to estimate uncertainty in the estimate of an association index. Click here for file

Additional File 8 **Figure_S7.pdf. Maximum modularity *Q*_max _as a function of the SRI threshold**. Data for each of the seasonal data sets correspond to the network structure curves shown in Figure 8 in the main text (see Methods in the main text for details). Click here for file

Additional File 9 **Figure_S8.pdf. Detecting differences in the shape of network structure curves**. *P*-value for the Mann-Whitney test of the comparison between the distribution of the network structure curve increments within window *w *before each SRI threshold with the corresponding distribution within the window *w *after that threshold are shown. Different colors correspond to different window sizes, as indicated. Dashed line shows the critical *P*-value of 0.05. Click here for file
