# Supplementary figures and images for: The dynamics of social networks among female Asian elephants
Source: BMC Ecol. 2011 Jul 27;11:17. doi: 10.1186/1472-6785-11-17 (PMC3199741; doi:10.1186/1472-6785-11-17)

SRI

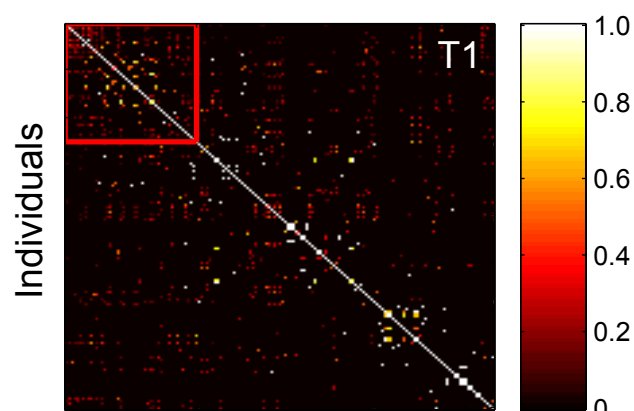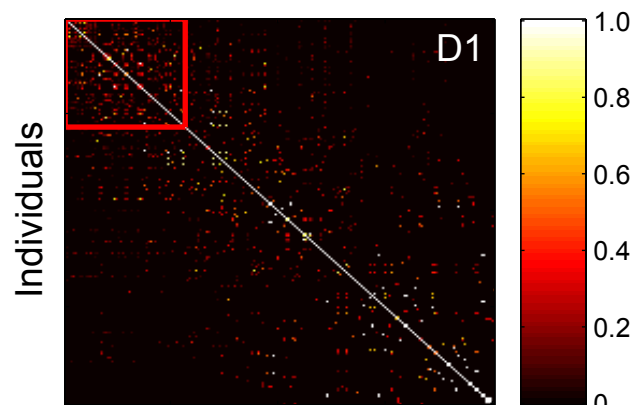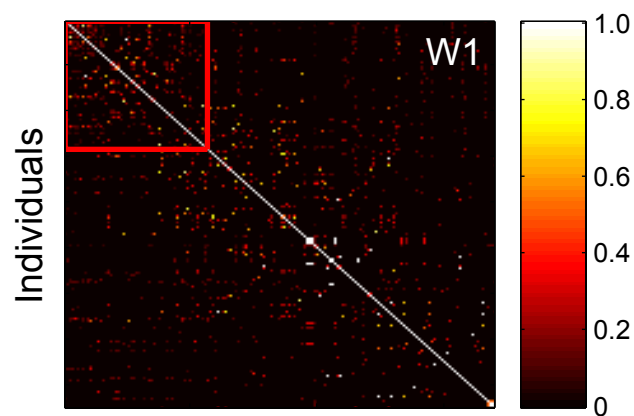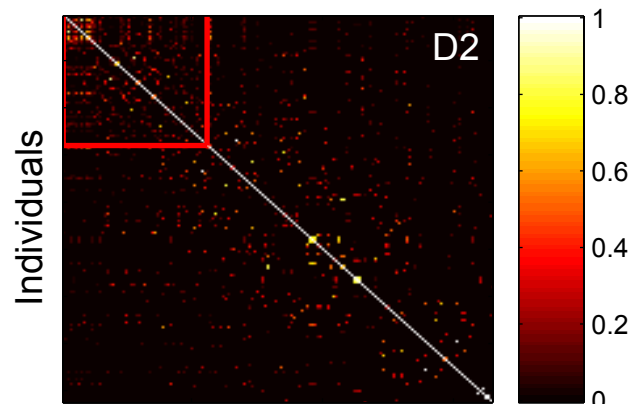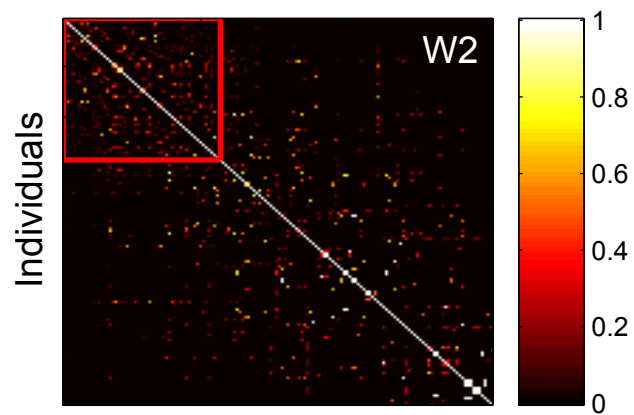

Uncertainty

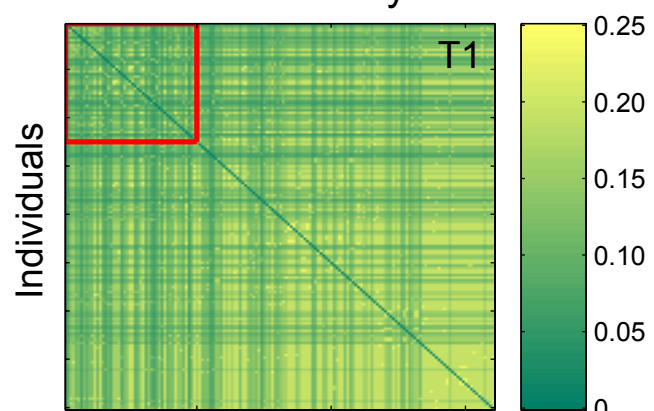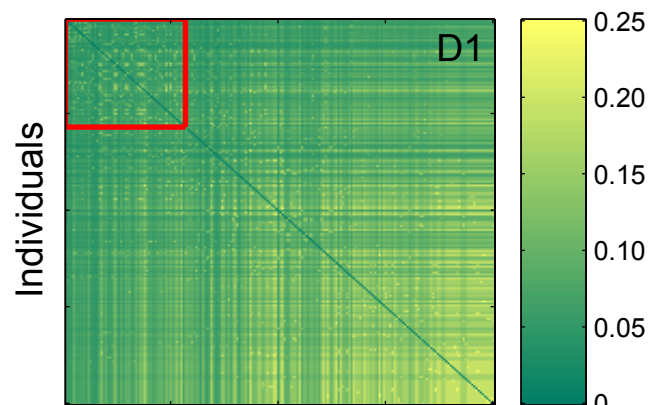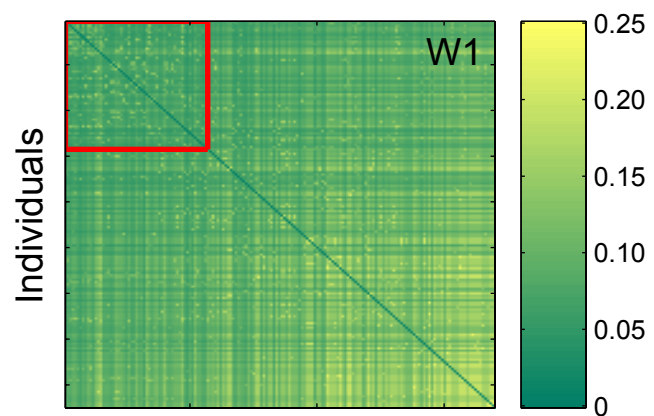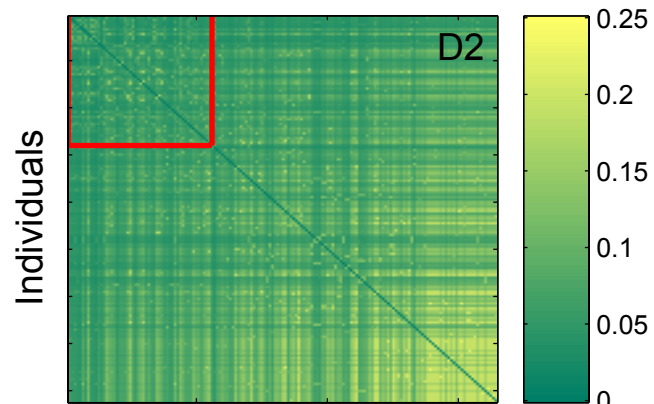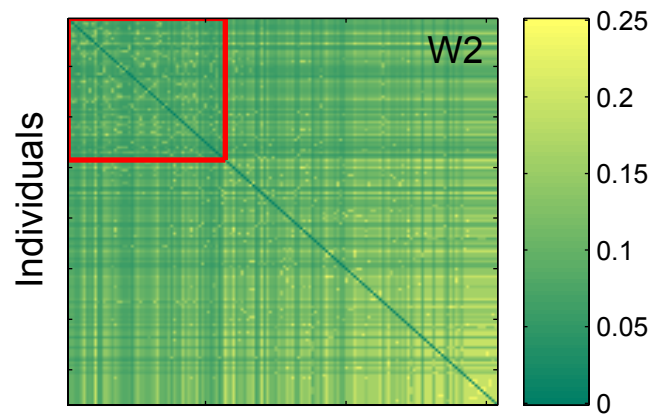

Supplement: Additional File 1 — Figure_S1.pdf. Association index matrices and matrices of uncertainty by season. SRI matrices (left column) and the matrices of uncertainty measure based on expression [S1] in the supplementary text (right column). See additional File 7 for supplementary text. x- and y- axes represent individuals, ordered by the total number of sightings across the whole study period. Red squares show the individuals with 30 or more sightings. [file 1472-6785-11-17-S1.PDF]

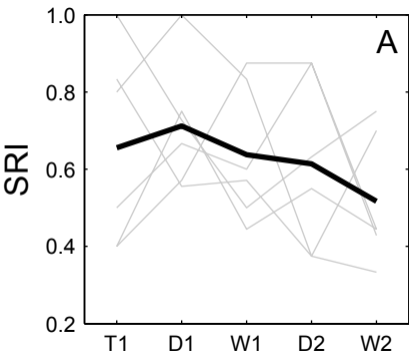

Supplement: Additional File 2 — Figure_S2.pdf. Temporal SRI trajectories of type A. Type A trajectories are relationships that are maintained at SRI values above 0.3 in all seasons, and hence exemplify stable associations. [file 1472-6785-11-17-S2.PDF]

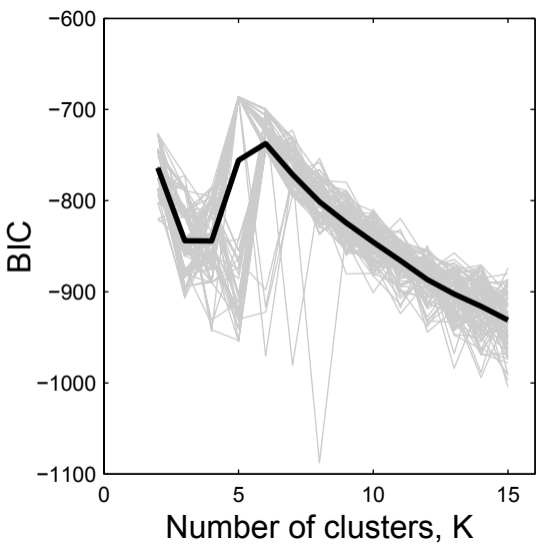

Supplement: Additional File 3 — Figure_S3.pdf. Determining the optimal number of clusters for clustering temporal SRI trajectories. Bayesian Information Criterion (BIC) as a function of the number of clusters, K, into which the temporal SRI trajectories are partitioned by the K-means algorithm. Light gray curves represent 100 K-means clustering runs with random initial conditions. Thick black curve represents the mean over these runs. The mean BIC curve peaks at K = 6 indicating that there are 6 typical shapes of temporal SRI trajectories. [file 1472-6785-11-17-S3.PDF]

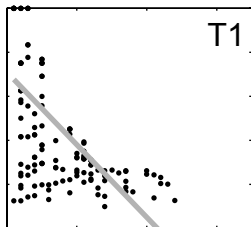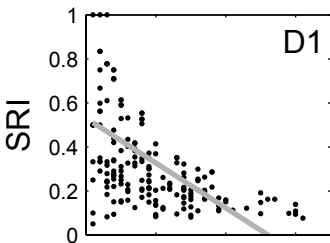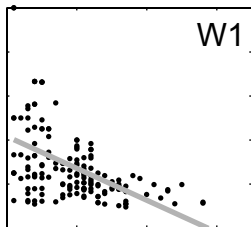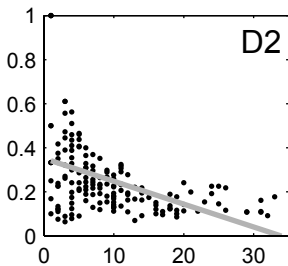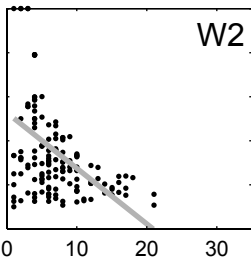

Number of associates

Supplement: Additional File 4 — Figure_S4.pdf. Trade-off between the number of associates and the strength of association by season. Each point shows the number of associates of a core individual (abscissa) and the average non-zero SRI value for that individual (ordinate). Data is displayed for each season separately (averages across seasons are shown in Figure 4 in the main text). [file 1472-6785-11-17-S4.PDF]

$T = 0.1$

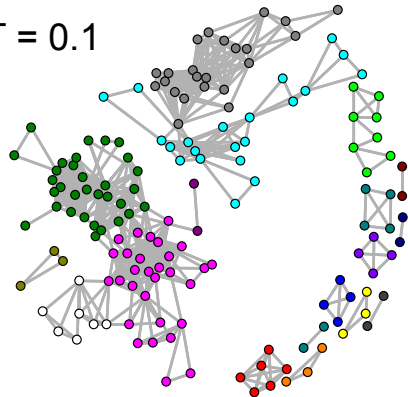

$T = 0.2$

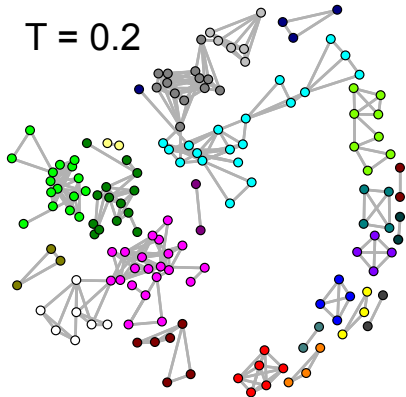

$T = 0.3$

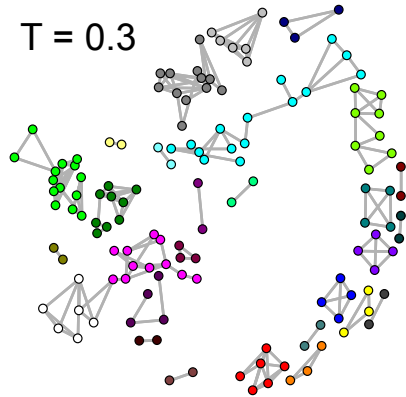

$T = 0.4$

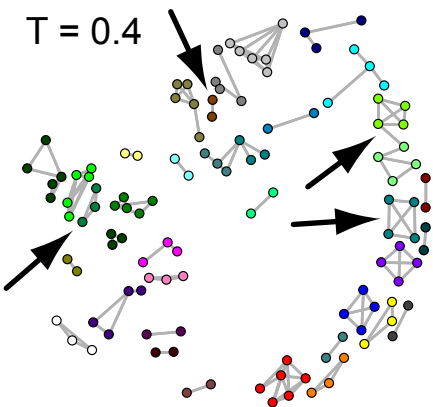

$T = 0.4$

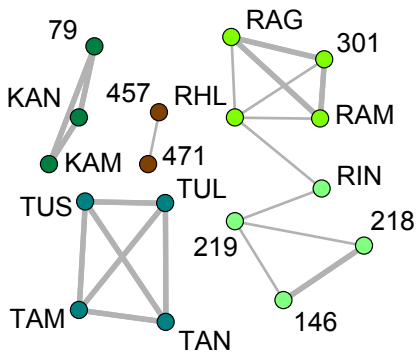

$T = 0.5$

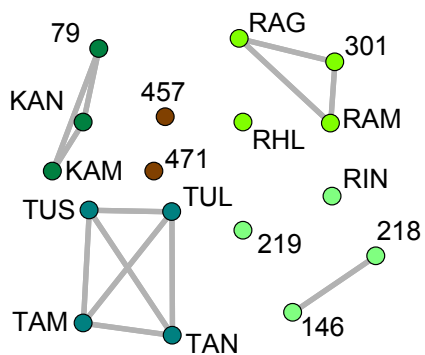

Supplement: Additional File 5 — Figure_S5.pdf. Girvan-Newman clusters for season T1 at various SRI thresholds. This figure is analogous to the schematic shown in Figure 7 in the main text. Corresponding network structure curves are shown in Figure 8 of the main text. Individuals that do not have ties at or above the threshold value are removed for the sake of clarity. Arrows indicate groups enlarged in the last two panels. Cluster colors correspond to Figure 5 in the main text. Maximum separation of groups occurs in this season at SRI values above 0.4. Beyond the SRI threshold of 0.6 most of these clusters also degenerate. [file 1472-6785-11-17-S5.PDF]

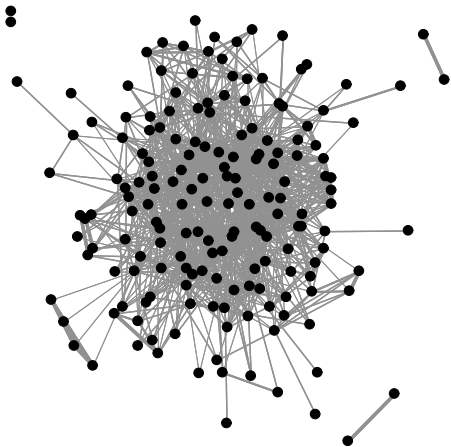

Supplement: Additional File 6 — Figure_S6.pdf. Social network for randomized T1 data. An example of a social network generated by the randomization procedure described in the methods. The network is relatively homogenous, without distinct clusters. [file 1472-6785-11-17-S6.PDF]

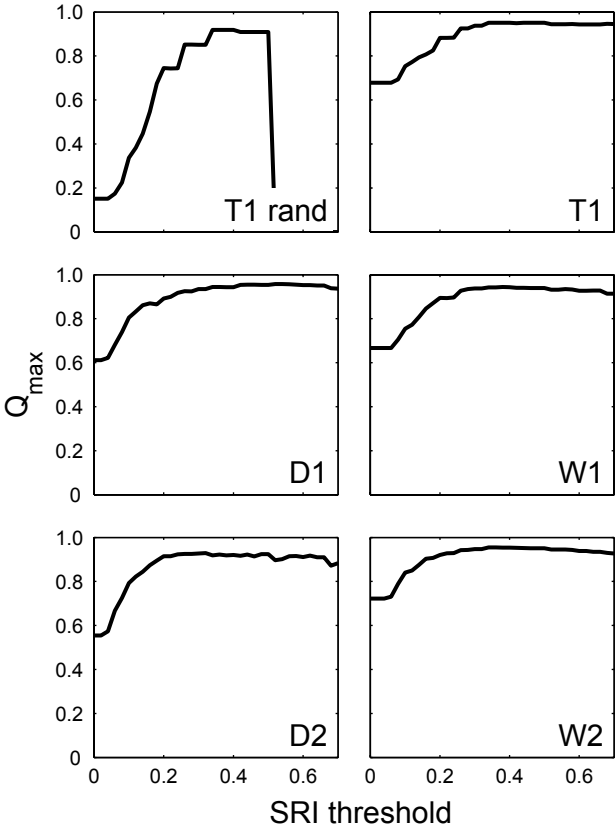

Supplement: Additional File 8 — Figure_S7.pdf. Maximum modularity Qmax as a function of the SRI threshold. Data for each of the seasonal data sets correspond to the network structure curves shown in Figure 8 in the main text (see Methods in the main text for details). [file 1472-6785-11-17-S8.PDF]

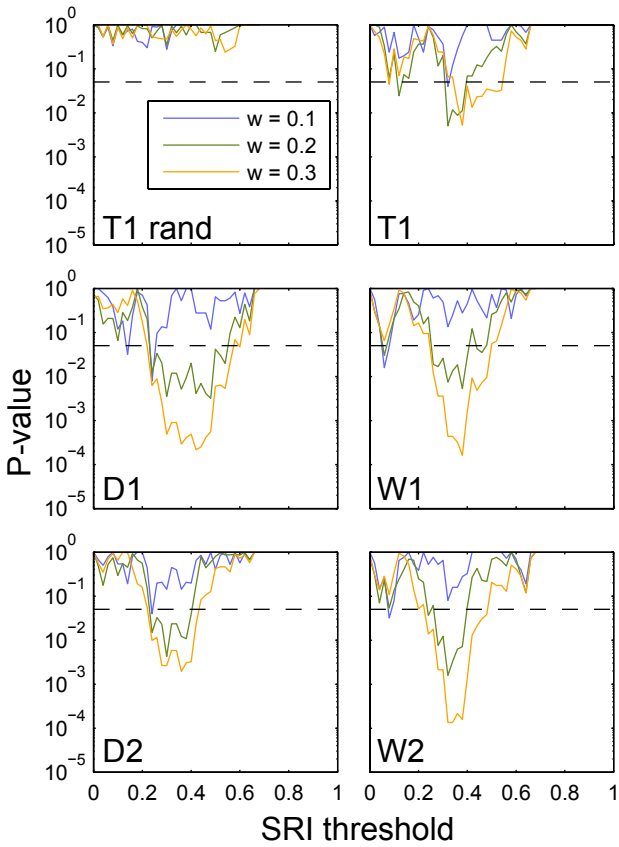

Supplement: Additional File 9 — Figure_S8.pdf. Detecting differences in the shape of network structure curves. P-value for the Mann-Whitney test of the comparison between the distribution of the network structure curve increments within window w before each SRI threshold with the corresponding distribution within the window w after that threshold are shown. Different colors correspond to different window sizes, as indicated. Dashed line shows the critical P-value of 0.05. [file 1472-6785-11-17-S9.PDF]
