# Supplementary material for: The dynamics of social networks among female Asian elephants
Source: BMC Ecol. 2011 Jul 27;11:17. doi: 10.1186/1472-6785-11-17 (PMC3199741; doi:10.1186/1472-6785-11-17)
Supplement: Additional File 7 — Supplementary_Text.doc. Description of the procedure to estimate uncertainty in the estimate of an association index. [file 1472-6785-11-17-S7.PDF]

The Dynamics of Social Networks in Asian Elephants

S. de Silva, A.D.G. Rangeewa, and S. Kryazhimskiy

Supplementary Text

Uncertainty in association indices

To assess the degree of uncertainty in our estimates of association indices, we derived an expression that is similar to that proposed by Whitehead [S1], but is based on a Bayesian argument. The details of this analysis will be described elsewhere. Briefly, if individuals A and B are together during an observation with probability α, then out of N observations (in which either A and/or B are observed), they will be observed together with the binomial probability with parameters α and *N*. Assuming, conservatively, a uniform prior on α, the posterior distribution of α is a beta-distribution with parameters $X+1$ and $N-X+1$. The mean of the posterior distribution, $\hat{\alpha}=\frac{X+1}{N+2}$, could be used as an estimate of the association index (although we use the standard estimator, X/N, in this paper). We use the standard deviation of the posterior distribution,

$$S=\sqrt{\frac{\hat{\alpha}(1-\hat{\alpha})}{N+3}}, \qquad \text{[S1]}$$

as a measure of uncertainty of the SRI estimate. The association index matrices as well as corresponding values of *S* are shown in Figure S1.

References

S1. Whitehead H: **Precision and power in the analysis of social structure using associations.** *Anim. Behav.* 2008, **75**:1093-1099
